# Comparative Impact of Alternate-Day Fasting and Time-Restricted Feeding on Placental Function and Fetal Development in Maternal Obesity

**DOI:** 10.3390/nu17010025

**Published:** 2024-12-25

**Authors:** Siyuan Liu, Lun Hua, Xi Mo, Bing Lei, Ruihao Zhang, Shihao Zhou, Xuemei Jiang, Zhengfeng Fang, Bin Feng, Lianqiang Che, Shengyu Xu, Yan Lin, De Wu, Yong Zhuo, Chao Jin

**Affiliations:** 1 Animal Nutrition Institute, Sichuan Agricultural University, Chengdu 611130, China; 18384366502@163.com (S.L.); hualun@sicau.edu.cn (L.H.); 13765493262@163.com (X.M.); 18990972642@163.com (B.L.); zrhhhh13@163.com (R.Z.); 18190974087@163.com (S.Z.); 71310@sicau.edu.cn (X.J.); zfang@sicau.edu.cn (Z.F.); fengbin@sicau.edu.cn (B.F.); che.lianqiang@sicau.edu.cn (L.C.); shengyuxu@sicau.edu.cn (S.X.); linyan@sicau.edu.cn (Y.L.); wude@sicau.edu.cn (D.W.); zhuoyong@sicau.edu.cn (Y.Z.); 2 Key Laboratory for Animal Disease-Resistant Nutrition of Sichuan Province, The Ministry of Education of China, Chengdu 611130, China

**Keywords:** alternate-day fasting, fetal growth restriction, maternal obesity, placenta, time-restricted feeding

## Abstract

Background: Maternal obesity detrimentally affects placental function and fetal development. Both alternate-day fasting (ADF) and time-restricted feeding (TRF) are dietary interventions that can improve metabolic health, yet their comparative effects on placental function and fetal development remain unexplored. Objectives: This study aims to investigate the effects of ADF and TRF on placental function and fetal development during maternal consumption of a high-fat diet (HFD). Methods: One hundred 8-week-old female mice were assigned to one of four dietary regimens: (1) normal diet with ad libitum feeding (NA); (2) HFD with ad libitum feeding (HA); (3) HFD with ADF (HI); and (4) HFD with TRF (HT), administered both before and during pregnancy. On gestational day 18.5, serum and placental samples were collected from both mothers and fetuses to examine placental function and fetal development. Results: During gestation, both ADF and TRF substantially alleviated the metabolic impairments caused by an HFD in obese maternal mice. TRF mice demonstrated enhanced placental nutrient transport and fetal development, associated with reduced endoplasmic reticulum (ER) stress and inflammatory responses. In contrast, ADF markedly intensified placental stress and inflammatory responses, diminished placental nutrient transport efficiency, and consequently induced fetal growth restriction. Conclusions: Both ADF and TRF during pregnancy significantly mitigated metabolic impairments in obese dams on an HFD. TRF mice demonstrated enhanced placental nutrient transport and fetal development, associated with reduced endoplasmic reticulum (ER) stress and inflammatory responses. In contrast, ADF markedly intensified placental stress and inflammatory responses, diminished placental nutrient transport efficiency, and consequently induced fetal growth restriction.

## 1. Introduction

In recent years, the prevalence of obesity has increased rapidly across various nations [1]. Given the central role of women in society, the health issues pertaining to women warrant particular attention [2]. The deleterious effects of maternal consumption of a high-fat diet (HFD) on the intrauterine environment and fetal development are well-established [3,4]. Lipotoxicity, usually resulting from HFD, leads to increased placental stress and inflammation [5,6]. The placenta, as the crucial interface between the mother and fetus, plays an essential role in regulating nutrient and gas exchange to support a successful pregnancy [7]. A growing body of evidence indicates that maternal obesity significantly alters placental function [8,9]. Mechanistically, maternal obesity induces apoptosis in placental trophoblasts by generating reactive oxygen species (ROS) and causing endoplasmic reticulum (ER) stress [10,11]. Additionally, the increase in cellular stress is linked to the production of inflammatory cytokines in the placenta, triggered by HFD [5]. Given the frequent association between obesity and placental dysfunction, non-pharmacological interventions targeting both obesity and placental damage have become a critical focus of research.

Intermittent fasting (IF) has gained widespread recognition as a dietary regimen, emerging as one of the most innovative and promising strategies for weight management and the treatment of metabolic disorders [10,12]. IF typically includes methods such as time-restricted feeding (TRF), alternate-day fasting (ADF), and the 5:2 diet. The 5:2 diet entails consuming a regular diet for five days of the week while limiting calorie intake to 500–600 calories on the remaining two days [13]. Specifically, IF has been shown to significantly reduce levels of leptin, total cholesterol, low-density lipoprotein, and triglycerides in obese women while also decreasing body weight and fat content [14,15,16]. Remarkably, even in the context of a high-fat diet, IF has been shown to substantially improve lipid profiles in obese individuals [17,18]. Furthermore, animal studies have revealed that IF can reduce fasting blood glucose and insulin levels in obese mice [19].

Moreover, IF has been demonstrated to have significant impacts on reproductive functions. IF has been observed to improve oocyte fertilization rates and promote blastocyst formation in aged female mice, resulting in an increased litter size [20]. Our previous research has demonstrated that TRF enhances ovarian follicle development and effectively mitigates HFD-induced embryonic loss, thereby ameliorating the defects in luminal closure observed on the 4.5th day of gestation [21,22]. Compared to ad libitum feeding regimens, TRF significantly suppresses HFD-induced apoptosis and inflammatory signaling in rat placental tissues [23]. At the molecular level, TRF markedly restricted the upregulation of oxidative and endoplasmic reticulum stress, as well as the disruption of autophagic flux, all of which are induced by HFD in rat placental tissue, in contrast to ad libitum feeding [10].

While IF has the potential to enhance reproductive performance, its effects are not universally beneficial, and outcomes can vary depending on the specific type of IF [24]. For instance, a study by Kumar S. et al. demonstrated that a 12-week intervention of ADF in female rats led to a significant reduction in serum estradiol and luteinizing hormone (LH) levels [25]. This hormonal decline was associated with a notable decrease in estrous cycle frequency and ovarian weight, suggesting that ADF may adversely affect the reproductive physiology of female rats. Alaa et al. found that fasting pregnant mice from 17:00 to 09:00 reduced the activity of placental System A transporters, which may contribute to restricted fetal growth [26]. Similarly, in prepubertal gilts subjected to an 8 h TRF regimen for 14 days, there was a reduction in follicle number and volume, as well as uterine weight, after just 7 days of TRF compared to ad libitum-fed controls [27]. In the context of pregnant Muslim women, research by Savitri AI et al. demonstrated fasting during Ramadan significantly reduced neonatal birth weight when practiced during early pregnancy, although no significant effects were observed during mid or late pregnancy [28]. The study conducted by Mirghani and Hamud identified several adverse outcomes associated with maternal fasting. These outcomes include a higher frequency of admissions to the special care baby unit, an increased risk of developing gestational diabetes mellitus, and a greater likelihood of labor induction [29]. These findings indicate that the impact of intermittent fasting on maternal reproductive capacity is complex and not fully understood, underscoring the need for further systematic research to comprehensively explore its effects.

This study aims to evaluate the impact of ADF and TRF on the metabolic health and reproductive performance of obese mothers subjected to a high-fat diet and to investigate whether these dietary patterns can mitigate the detrimental effects of a high-fat diet on placental morphology, function, and fetal development.

## 2. Methods

### 2.1. Materials and Methods

This study was conducted in strict accordance with the Standard Operating Procedures for Laboratory Animal Use at Sichuan Agricultural University. All animal-related procedures received approval from the Institutional Animal Care and Research Committee of Sichuan Agricultural University under the animal care authorization number SI20220092.

### 2.2. Animals and Feeding Schedule

**Figure 1:**
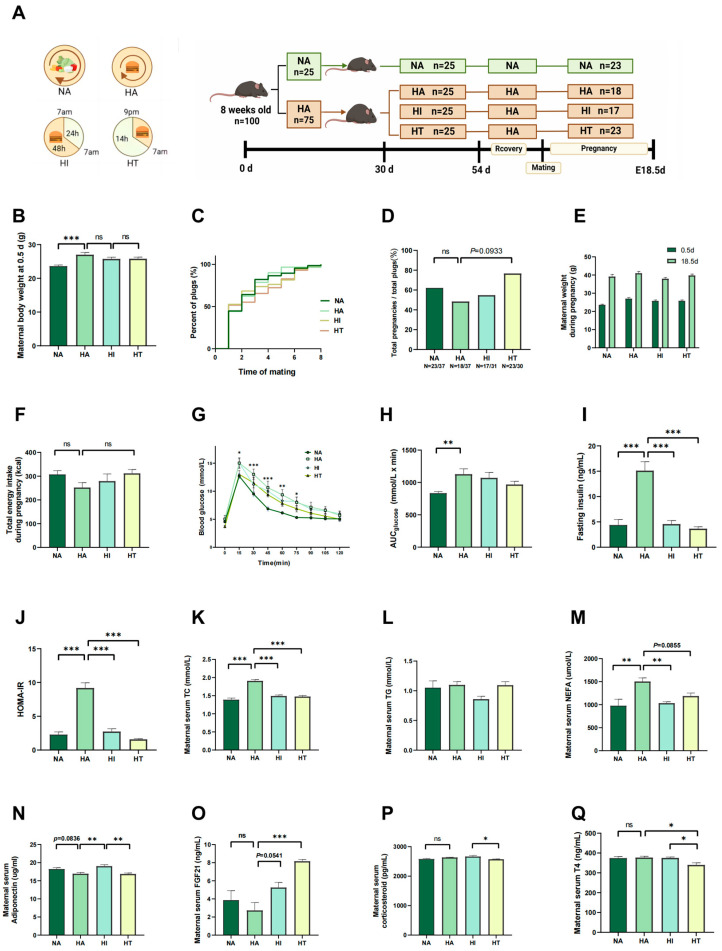
Impacts of intermittent fasting on maternal metabolism. (**A**) Flow chart illustrating the allocation of mice and treatments. (**B**) Body weight on the day of vaginal plug. (**C**) Percentage of mice with a vaginal plug. (**D**) Ratio of total pregnancies to total plugs. The denominator represents the total number of plugs observed in the group during the mating phase, while the numerator represents the mice that were successfully pregnant in the end. (**E**) Body weight at E0.5 d and E18.5 d. (**F**) Total energy intake during gestation. NA (*n* = 12), HA (*n* = 12), HI (*n* = 13), HT (*n* = 13) for (**B**,**E**,**F**). (**G**) Blood glucose. Measure blood glucose every 15 min. * *p* < 0.05, ** *p* < 0.01, and *** *p* < 0.001 between the HA and NA groups. (**H**) Area Under the Curve. (**I**) Fasting insulin in maternal mice at E18.5. (**J**) Calculated insulin resistance following 10 h of fasting in maternal mice at E18.5. (**K**–**M**) Maternal blood lipid levels at E18.5, including triglycerides, total cholesterol, and non-esterified fatty acids. (**N**–**Q**) Maternal hormone levels, including adiponectin, fibroblast growth factor 21, corticosteroid, and thyroid hormone 4. NA (*n* = 6), HA (*n* = 6), HI (*n* = 6), and HT (*n* = 6) for (**G**–**Q**). Data are expressed as the mean ± S.E. * *p* < 0.05, ** *p* < 0.01, and *** *p* < 0.001.

Eight-week-old female C57BL/6N mice were obtained from Vital River Laboratory Animal Technology Co., Ltd. (Beijing, China). A total of 100 mice were randomly divided into two groups: the NA group (*n* = 25), which was fed a normal diet (ND) ad libitum, and the HA group (*n* = 75), which was fed an HFD ad libitum. Mice were housed in individual ventilated cages, with each cage containing five mice. Mice were maintained with a light cycle of 12 h on and 12 h off, specifically from 8:00 A.M. to 8:00 P.M. The mice were fed the ND or HFD for 30 days, and then the HA mice were further randomly divided into three subgroups, with 25 mice per subgroup, for 24 days: HA, which continued with ad libitum feeding on the HFD; HI, an ADF regimen in which the mice underwent a cycle of 24 h fasting followed by 48 h of ad libitum feeding on the HFD; and HT, a TRF regimen in which the mice had access to food from 21:00 to 07:00 but fasted for the remainder of each day. A schematic of the feeding protocols used in this study is shown in Figure 1A. Mice in the TRF and ADF groups were transferred to cages containing no food but with free access to water during the fasting period. The temperature in the animal housing room is maintained between 22 °C and 24 °C.

The ND, sourced from Beijing Keao Xieli Feed Co., Ltd. (Beijing, China), consisted of 65.08% carbohydrates, 23.07% protein, and 12% fat. The HFD, purchased from Research Diets (#D12492) (New Brunswick, NJ, USA), contained 20% carbohydrates, 20% protein, and 60% fat. The energy content is 3.40 kcal/g for ND and 5.420 kcal/g for HFD, respectively. Detailed diet compositions were performed as previously described [21].

### 2.3. Fertility Assessment

After 24 days on the HA, HI, and HT dietary regimens, the mice were transitioned back to the HA regimen for 5 days to mitigate stress related to abrupt fasting during mating and early pregnancy. Fifty confirmed fertile male C57BL/6N mice, approximately 3 to 4 months old and maintained on ND, were used for mating with female mice. Male mice were then cohoused with female mice in a 1:1 ratio at 21:00, and they remained together until 07:00 the next morning. In this study, female mice were paired with fertile males for eight days, during which the presence of vaginal plugs was monitored and documented. The ratio of successful pregnancies to the number of vaginal plug observations was calculated for each group. The day on which a vaginal plug was detected was recorded as day 0.5 of pregnancy (E0.5). Following the detection of a vaginal plug, the female mice were housed individually. Mice that have not shown plugs after eight mating attempts were culled.

On gestational day 18.5 (E18.5), the pregnant mice, 12–13 mice per group, were euthanized using carbon dioxide, and maternal serum and placental samples were collected for further analysis. The mouse uterus was surgically removed and meticulously dissected to evaluate the condition of the fetuses. Viable fetuses were identified by their ruddy complexion and noticeable respiratory activity, while non-viable fetuses displayed a pallid coloration and flaccid musculature.

### 2.4. Glucose Tolerance Tests

Six mice per group underwent a fasting period beginning at 9:00 P.M. on gestational day 17.5. The following morning, gestational day 18.5, at 7:00 A.M., they were weighed and subsequently administered glucose intraperitoneally at a dose of 2 g/kg. Blood glucose levels were measured sequentially at intervals of 30, 60, 90, and 120 min, using blood samples obtained from the tail vein. Fasting and postprandial insulin levels were assessed by collecting blood samples from the tail both before and 30 min after glucose injection. Insulin resistance was calculated using the homeostatic model assessment for insulin resistance (HOMA-IR) formula: HOMA-IR [fasting insulin in μU/mL × (fasting blood glucose in mg/dL^−1^ × 0.055)/22.5].

### 2.5. Serum Biochemistry Hormone Measurements

Six serum samples were selected from each group for testing. Serum levels of triglycerides (TG), total cholesterol (TC), and non-esterified fatty acids (NEFA) were measured using an automatic biochemical analyzer (3100; Hitachi, Tokyo, Japan). Adiponectin (EDMADP; Millipore, Burlington, MA, USA), insulin (PTG-KE10089-96T; Proteintech, Rosemont, IL, USA), T3 (EIAT3C; Thermo, Waltham, MA, USA), T4 (EIAT4C; Thermo), and corticosteroid (ADI-900-097; Enzo Life Sciences, Farmingdale, NY, USA) concentrations in serum were determined using commercial ELISA kits, following the manufacturer’s instructions.

### 2.6. Quantitative Real-Time PCR (RT-PCR) Analysis

For RT-PCR analysis, a placenta was selected from each litter of every dam (*n* = 8) with a similar number of fetuses and ensured that the placenta weight was close to the average value in that litter. Total RNA was extracted from placental tissue using TRIzol reagent (Thermo Fisher Scientific, Waltham, MA, USA) and further purified with RNA mini-columns (Takara Bio, Kusatsu, Japan). cDNA was synthesized from 1 μg of RNA (AT341; TransScript) (Beijing, China). RT-PCR was performed in a 10 μL reaction volume using SYBR Green (Takara Bio). Gene expression levels were normalized to β-actin expression levels. The cycle threshold (2^−ΔΔCT^) method was used to calculate the relative gene expression. The primer sequences for the target genes (*Slc2a1*, *Slc2a3*, *Slc2a4*, *Slc2a5*, *Slc5a1*, *Slc27a1*, *Slc7a6*, *FABP3*, *FABP4*, *Slc1a1*, *Slc7a1*, *Slc38a1*, *Slc38a4*, *Col1α1*, *Col1α2*, *Col3α1*, *Col4α5*, *F4/80*, *Mcp1*, *Tnfα*, *IL1β*, *IL6*, *Arg1*, *Mgl1*, *iNOS*, *Mrc2*, *IL10*, *GRP78*, *Chop*, *XBP1s*, *IRE1α*, *Perk*, and *ATF6*) are provided in Table S1 and Table S2.

### 2.7. Western Blot

In the WB analysis, placentas with weights close to the average value within a litter were selected. Three placentas were chosen from three dams in each group. Protein extraction and Western blotting were performed as previously described [30]. In brief, samples were homogenized in a lysis buffer (P0013B; Beyotime Institute of Biotechnology, Shanghai, China) containing a protease inhibitor cocktail (04693132001; Roche, Basel, Switzerland). The resulting homogenates were then centrifuged at 12,000× *g* for 30 min at 4 °C. Following centrifugation, the supernatant was collected, and protein concentrations were quantified using a BCA Protein Assay Kit (23227; Thermo Fisher Scientific) measured with a plate reader. For SDS-PAGE, proteins were denatured by boiling at 95 °C for 5 min and subsequently separated on a 10% gel. Proteins were transferred onto a polyvinylidene fluoride (PVDF) membrane (1620177; Bio-Rad Laboratories, Hercules, CA, USA). The membrane was washed with Tris-buffered saline containing Tween 20 (TBST) and blocked with 1% bovine serum albumin (BSA) in TBST at room temperature for 1 h under gentle agitation. Following blocking, the membranes were incubated overnight at 4 °C with the respective primary antibodies. The antibodies used were anti-GAPDH (#1E6D9), anti-GRP78 (#11587-1-AP) and anti-VEGFα (#19003-1-AP) from Proteintech, as well as anti-XBP1s (#D46F1), and anti-CHOP (#D46F1) from Cell Signaling Technology (Danvers, MA, USA).

### 2.8. Histological Analysis

Each pregnant dam selected a placenta with a weight close to the average for HE staining. Placental tissues were fixed in 4% paraformaldehyde in phosphate-buffered saline, then dehydrated, embedded in paraffin, and sectioned at a thickness of 5 μm. The sections were stained with hematoxylin and eosin or incubated with a CD31 antibody, followed by 3,3-diaminobenzidine staining, and examined using bright-field microscopy (Nikon 80i, Nikon, Minato City, Japan). For placental HE staining analysis, placentas (*n* = 11) from each group were selected for area analysis of the labyrinthine zone (LZ), junctional zone (JZ), and decidual zone (DZ). The areas of the LZ, JZ, and DZ of the placenta were quantified using NDP.view 2 software. Fluorescence imaging of the placenta was analyzed with ImageJ software (version 1.45).

### 2.9. Statistical Analysis

Initially, each of the NA, HA, HI, and HT groups comprised 25 mice. During the experiment, 2, 2, 1, and 1 female mice in these respective groups did not exhibit any vaginal plugs and were consequently excluded from further analysis. After observing plugs, the corresponding dietary intervention was immediately implemented. Infertile mice that observed plugs multiple times but did not become pregnant were also eliminated, with no eliminations from the NA group, 5 from the HA group, 7 from the HI group, and 1 from the HT group. This left 23 females in the NA group, 18 in the HA group, 17 in the HI group, and 23 in the HT group who successfully became pregnant. On embryonic day 18.5 (E18.5), tissue sampling required the sacrifice of 12 mice from the NA group, 12 from the HA group, 13 from the HI group, and 13 from the HT group. Our original intention was to examine the impact of maternal dietary regimens on offspring metabolic status. However, after sacrificing 12 HA and 13 HI pregnant mice, which would leave a small number of mice (*n* = 4) in HI groups, we had to abandon this objective.

Data were analyzed using GraphPad Prism 6 software. The normality was assessed using the Shapiro–Wilk test and Kolmogorov–Smirnov test. When the assumption of normality is not met (for stillbirth), non-parametric methods are used as alternatives to parametric methods, such as the Kruskal–Wallis test. For comparisons involving more than two groups, a one-way analysis of variance (ANOVA) was performed, followed by the Student’s *t*-test for multiple comparisons to identify significant differences. Data are presented as mean ± standard error of the mean. Statistical significance was determined at a threshold of *p* < 0.05.

## 3. Results

### 3.1. Impacts of Intermittent Fasting on Maternal Metabolism

An overview of the experimental schematic for diet assignment is presented in Figure 1A. At the start of the experiment, there was no significant difference in the body weight of 8-week-old mice. However, after one month of a high-fat diet, the body weight of the high-fat group increased significantly compared to the mice on a normal diet (Figure S1A,B). The HFD mice in the HA, HI, and HT groups exhibited greater body weight than the NA mice, consistent with their increased caloric intake (Figure 1B). The breeding experiment showed no significant differences in the time to vaginal plug formation among the four experimental groups (Figure 1C). However, an upward trend in successful mating probability was observed in the TRF group compared to the HA group (*p* = 0.093, Figure 1D). During gestation, there were no significant differences in total energy intake or changes in body weight among the four experimental groups (Figure 1E,F).

Glucose tolerance tests (GTT) revealed that mice in the HA group exhibited greater insulin resistance, while those on HI or HT during pregnancy showed improved glucose tolerance (Figure 1G–J). As expected, a high-fat diet led to elevated lipid levels; however, biochemical analyses revealed that mice subjected to TRF and ADF demonstrated significant improvements in lipid profiles—including total triglycerides; total cholesterol; and free fatty acids—compared to their ad libitum HFD counterparts (Figure 1K–M). Additionally, adiponectin levels were significantly higher in the high-fat intermittent fasting (HI) group compared to both the HA and HT groups (Figure 1N).

Fibroblast Growth Factor 21 (FGF21) is pivotal in the modulation of metabolic processes and the enhancement of insulin sensitivity [31]. The serum FGF21 level in the HT mice was greater in contrast to the NA and HA groups (Figure 1O). However, the HI mice had elevated corticosterone levels than the HT mice (Figure 1P). Therefore, we further assessed maternal thyroxine (T4) concentrations, and the HT mice had significantly higher T4 levels (Figure 1Q).

### 3.2. Impacts of Intermittent Fasting on Fetal Development

**Figure 2:**
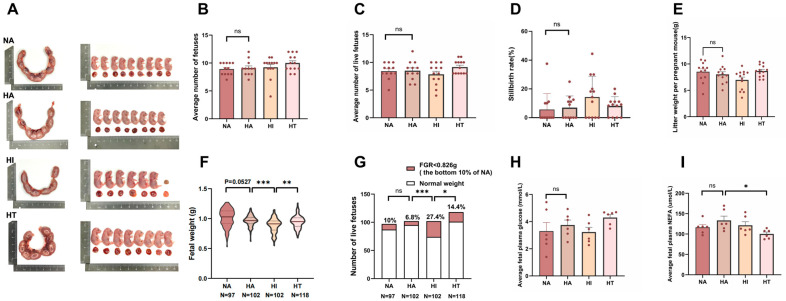
Impacts of intermittent fasting on fetal development. (**A**) Image of the fetus at day 18.5 of gestation. (**B**) Average number of fetuses. (**C**) Average number of live fetuses. (**D**) Fetal mortality at E18.5. (**E**) Litter weight. NA (*n* = 12), HA (*n* = 12), HI (*n* = 13), and HT (*n* = 13) for (**B**–**E**). (**F**) Fetal weight. (**G**) Those below the bottom 10% (0.826 g) of body weight in the normal group were considered Fetal Growth Restriction (FGR). (**H**,**I**) Average fetal plasma glucose and free fatty acid levels (*n* = 6). Data are expressed as the mean ± S.E. * *p* < 0.05, ** *p* < 0.01, and *** *p* < 0.001.

The number and morphology of fetuses at gestational day 18.5 were measured (Figure 2A). There were no significant differences in total litter size, stillbirths, live births, or stillbirth rates among the four experimental groups (Figure 2B–D). However, the HI mice had lower litter weight than the other groups (*p* = 0.061, Figure 2E). The HI mice had lower fetal weight than the other groups (Figure 2F). Fetal growth restriction (FGR) is a common pregnancy complication linked to long-term health issues such as impaired physical development, metabolic syndrome, and endocrine abnormalities [32]. Our study found that FGR was notably more pronounced in the HI group, while HT mice did not show similar adverse effects (Figure 2G). Fetal serum glucose and free fatty acid concentrations are crucial indicators of the fetus’s metabolic status. The fetal plasma glucose concentrations were significantly lower in mice subjected to HI, while HT resulted in improved fetal free fatty acid concentrations (Figure 2H,I).

### 3.3. Impacts of Intermittent Fasting on Placental Characteristics

**Figure 3:**
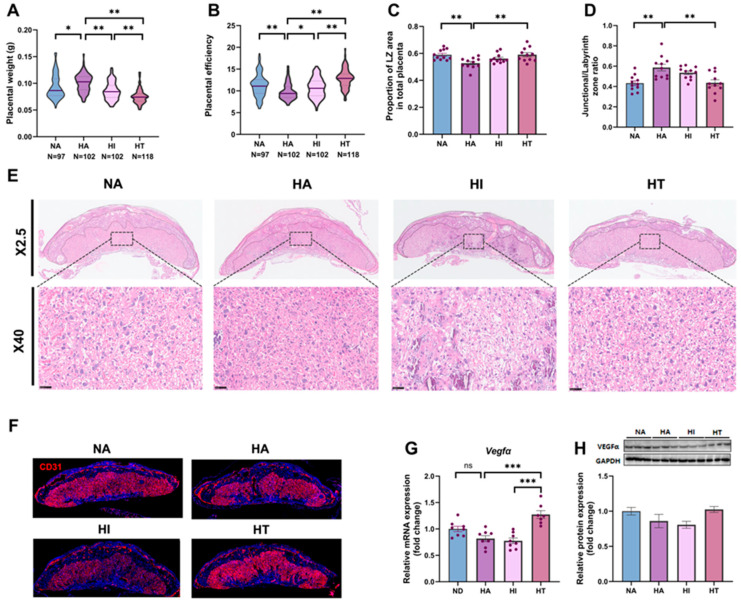
Impacts of intermittent fasting on placental characteristics. (**A**) The placenta weighs E18.5. (**B**) Placental efficiency is the ratio of fetal weight to placental weight. (**C**) Labyrinth zone (LZ) area of the placenta (*n* = 11). (**D**) Junctional/Labyrinth zone ratio (*n* = 11). (**E**) Images of the placental labyrinthine area enlarged X2.5 and X40. (**F**) CD31 immunofluorescence representative staining images. (**G**) mRNA relative levels of *Vegfα* in the placenta (GAPDH as the housekeeping control, *n* = 8). (**H**) Relative protein expression of VEGFα (GAPDH as control, *n* = 3). Data are expressed as the mean ± S.E. * *p* < 0.05, ** *p* < 0.01, and *** *p* < 0.001.

To better understand the factors contributing to reduced fetal weight, we conducted a detailed analysis of placental function [32]. The placental weight in HA mice is significantly higher than in the NA group but was decreased in the HI and HT groups (Figure 3A). The placental efficiency was lower in HA mice compared with the NA mice (Figure 3B). The placental efficiency of HI mice was greater than the HA mice but lower than the HT mice (Figure 3B). The placenta morphology is presented in Figure 3C–E. Figure 3E shows the overall histomorphology of placental HE staining across different treatment groups, emphasizing the morphological features of the junctional zone (JZ) and labyrinth zone (LZ). Histological examination via HE staining indicates a substantial reduction in the LZ area of the placenta in the HA group (Figure 3C). However, the ratio of the LZ to total placental area is significantly decreased in the HT group (Figure 3C), with a corresponding decrease in the JZ/LZ ratio (Figure 3D). To further explore the impact of feeding frequency on placental efficiency, we conducted an angiogenesis analysis of the placenta. Immunofluorescence analysis revealed a reduction in CD31 expression in the placenta, a marker of vascular formation and function, following intermittent fasting during pregnancy, indicating decreased vascular density (Figure 3F). Further analysis of VEGFα gene and protein levels showed enhanced angiogenesis in the HT group, while a decreasing trend was observed in the HI group (Figure 3G,H). These findings suggest that changes in feeding frequency can significantly influence placental function.

### 3.4. Impacts of Intermittent Fasting on Placental Expression Levels of Nutrient Transporters and Collagenases

**Figure 4:**
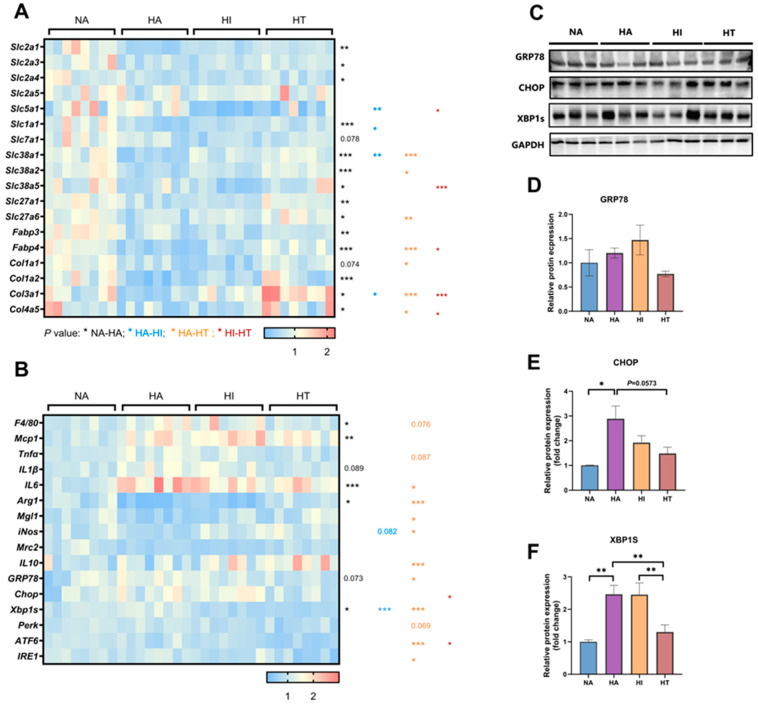
Time-restricted feeding prevents the adverse effects of a high-fat diet on placental function. (**A**) qRT-PCR analysis of glucose transporter gene (*Slc2a1*, *Slc2a3*, *Slc2a4*, *Slc2a5*, and *Slc5a1*), amino acid transporters (*Slc1a1*, *Slc7a1*, *Slc38a1*, and *Slc38a4*), fatty acid transporter (*Slc27a1*, *Slc27a6*, *Fabp3*, and *Fabp4*), and collagen (*Col1α1*, *Col1α2*, *Col3α1*, and *Col4α5*) expression levels in the placenta (*n* = 8). (**B**) qRT-PCR analysis of Macrophage type 1 gene (*F4/80*, *Mcp1*, *Tnfα*, *IL1β*, and *IL6*), Macrophage type 2 gene (*Arg1*, *Mgl1*, *iNos*, *Mrc2*, and *IL10*), and endoplasmic reticulum stress gene (*GRP78*, *Chop*, *Xbp1s*, *Perk*, *ATF6*, and *IRE1*) expression levels in the placenta (*n* = 8). (**C**–**F**) Protein expression levels of endoplasmic reticulum stress-related proteins (CHOP, XBP1s, and GRP78) in the placenta (*n* = 3). Data are expressed as the mean ± S.E. * *p* < 0.05, ** *p* < 0.01, and *** *p* < 0.001.

We measured mRNA expression levels of 15 nutrient transporters and 4 collagen (Figure 4A)**.** The expression levels of glucose transporters *Slc2a1* and *Slc2a3* are significantly reduced in the HFD-feeding groups (Figure 4A). Notably, the *Slc5a1* gene expression is significantly lower in the HI group compared to the HA and HT groups. However, the mRNA expression levels of placental *Slc2a4* and *Slc2a5* are unaffected by either a high-fat diet or IF interventions (Figure 4A). Compared to the control group, glutamate transporter *Slc1a1* expression is markedly reduced in the high-fat ad libitum diet group (Figure 4A). There is also a trend towards decreased expression of arginine and lysine transporters by HFD-feeding, such as *Slc7a1* (Figure 4A). The solute carrier family 38, which includes *Slc38a1, Slc38a2,* and *Slc38a5*, which are important for transporting neutral amino acids like alanine, serine, and glutamine, shows reduced expression in response to the HFD feeding compared to the NA mice (Figure 4A). HT does not have these adverse effects on the solute carrier family 38 (Figure 4A). The HFD feeding results in decreased expression of long-chain fatty acid transporters (*Slc27a1* and *Slc27a6*) and fatty acid-binding proteins (*FABP3* and *FABP4*). The mRNA expressions of *Col1α1* (*p* = 0.074), *Col1α2, Col3α1,* and *Col4α5* in NA mice were greater compared with the HA mice (Figure 4A), but the *Col3α1,* and *Col4α5* mRNA expressions were significantly elevated by HT compared with the HA and HI mice (Figure 4A).

### 3.5. ADF Exacerbates Inflammatory Responses and Endoplasmic Reticulum Stress in the Placenta, While TRF Does Not Elicit These Detrimental Effects

To further investigate the factors contributing to placental dysfunction, we analyzed the expression levels of macrophage-related markers in the placenta. Placental macrophages play a crucial role in both placental and fetal health [33]. They are not only involved in immune defense but also play key roles in tissue repair, angiogenesis, and nutrient transport [34]. A high-fat diet increases the expression of the M1 marker gene while decreasing the expression of the M2 marker gene, leading to exacerbated placental inflammation. (Figure 4B) Moreover, TRF significantly improves the imbalance of macrophages induced by a high-fat diet, resulting in an increased expression of the M2 marker gene, which can promote the formation and development of placental blood vessels by secreting angiogenic factors such as vascular endothelial growth factor (VEGF), thereby ensuring adequate blood supply to the fetus [35]. Therefore, TRF may enhance placental angiogenesis by improving the homeostasis of placental macrophages.

To further investigate whether IF induces Endoplasmic reticulum stress and subsequently affects placental development, we analyzed the expression levels of endoplasmic reticulum (ER) stress-related mRNA and proteins in the placenta (Figure 4B–F). Compared to the NA group, the HA group exhibited a significant increase in the expression of ER stress-related mRNA (*Xbp1s*) and proteins (CHOP and XBP1s), while HT treatment markedly decreased the ER stress-related genes (*GRP78, Xbp1s, ATF6*, and *IRE1*) compared with the HA mice.

Through the application of TRF, the expression of M2 and endoplasmic reticulum stress markers can be reduced, thereby mitigating the stress and inflammatory responses in placental cells and subsequently enhancing fetal health and development.

## 4. Discussion

Controlling obesity in pregnant women is crucial for improving pregnancy outcomes. Reproductive health is closely intertwined with energy homeostasis, with both obesity and rapid weight loss potentially leading to reproductive dysfunction [36,37,38]. TRF and IF are popular dietary strategies that have demonstrated effectiveness in preventing obesity and associated metabolic disorders [39]. This study aims to explore the effects of different feeding frequencies during pregnancy on maternal metabolism, fetal development, and placental function, focusing on the roles of ADF and TRF. The current findings show that adjusting the dietary ADF regimen during gestation significantly influences maternal metabolic health. In particular, TRF enhances the placenta’s nutrient absorption and transfer capacity, boosts collagen activity, and modulates the expression of inflammatory factors. These changes improve the placenta’s ability to support fetal growth and development more efficiently. The results provide valuable insights into dietary management during pregnancy, offering a scientific basis for promoting maternal and fetal health through improved placental function under maternal obesity.

Maternal obesity is characterized by alterations in circulating levels of various physiological substances, including hormones, nutrients, growth factors, cytokines, and lipids. This complex metabolic environment induced by obesity is closely associated with adverse pregnancy outcomes [40,41]. Previous research has shown that ADF results in food intake comparable to ad libitum feeding [42], yet it effectively improves lipid profiles in obese individuals [17,18]. In this study, we observed that while mice subjected to both TRF and ADF regimens exhibited similar caloric intake and weight gain as those with unrestricted feeding, their metabolic responses were markedly different. Our research further reveals that a high-fat diet not only elevates lipid levels in mice but also induces insulin resistance, both of which can be effectively mitigated through ADF and TRF. In a study on mice, those subjected to ADF exhibited significantly reduced fasting glucose and insulin levels compared to pair-fed groups [19]. Furthermore, plasma corticosterone, a biomarker of stress, has been shown to increase under high-fat diet conditions [43].In this study, we find no significant differences in T4 and corticosterone levels between the high-fat diet group and the normal diet group. However, stress hormone levels, such as corticosterone, are significantly higher in mice subjected to ADF compared to those in the TRF group. This suggests that ADF may more readily induce stress responses in mice. Our research findings indicate that the optimization of dietary intermittent fasting regimens can effectively improve blood glucose homeostasis in obese mothers. Consequently, these improvements reduce the risk of gestational diabetes and other metabolic disorders in obese female mice during pregnancy. Such metabolic enhancements not only contribute to the health maintenance of obese pregnant mothers but also establish a more stable nutritional environment for the developing fetus.

Maintaining an appropriate dietary pattern plays a crucial role in enhancing fetal growth and development [44]. Although a high-fat diet is typically associated with an increased risk of macrosomia in offspring, our study did not observe this outcome in the high-fat diet with ad libitum feeding. This discrepancy may be attributed to the reduced mitochondrial content observed in the placentas of obese women, a characteristic that diminishes the placenta’s *β*-oxidation capacity [45]. As a result, lipid accumulation occurs, and lipid transport to the developing fetus is diminished, thereby reducing the risk of excessive fetal adiposity. Our findings suggest that adopting a TRF approach during pregnancy can effectively lower the incidence of fetal growth restriction, ensuring that the fetus receives balanced nutrition in utero, which supports healthy development. Additionally, elevated free fatty acid levels are potentially linked to maternal metabolic disorders such as diabetes. Notably, TRF effectively mitigates the rise in free fatty acid levels in fetal mice, further promoting maternal and fetal health during pregnancy.

The placenta not only serves as an essential intermediary for nutrient and gas exchange but also plays a critical role in immune protection, hormone secretion, and waste elimination [46]. Consequently, the integrity of placental function is essential for fetal health. One important metric for assessing placental function is its efficiency, which is typically evaluated by the fetal-to-placental weight ratio. A higher ratio indicates greater placental efficiency, reflecting optimal nutrient transport to the fetus, while a lower ratio suggests suboptimal nutrient transport efficiency, potentially compromising fetal growth and development [47]. The study conducted by Hayes et al. demonstrated that the fetal-to-placental weight ratio in the obesity group was significantly lower than that of the control group, indicating impaired placental transport efficiency [48]. In our study, we observe that the fetal-to-placental weight ratio in the HA group is considerably lower than in the NA group, indicating reduced placental efficiency under a high-fat diet. However, the ratios in the HI and HT groups are significantly higher than in the HA group. This suggests that both ADF and TRF have the potential to enhance placental efficiency, even in the context of a high-fat diet, thereby improving nutrient transport to the fetus and supporting healthier fetal development.

The junctional zone of the placenta, particularly the intermediate layer of the central stratum, represents a pivotal region for nutrient transfer. The proportional area of this zone is positively correlated with the effectiveness of nutrient transport [49]. The variation in the area of the placental junctional zone is significantly influenced by dietary regimens. Vascular Endothelial Growth Factor (VEGF) plays a crucial role in nutrient transfer by promoting angiogenesis within the placental chorion and stroma [50]. When VEGF binds to its receptors (VEGFR), it activates downstream signaling pathways that facilitate the formation of new blood vessels, thereby ensuring an adequate blood supply to the fetus [51]. Evidence suggests that a deficiency or inhibition of VEGF can lead to insufficient placental angiogenesis, resulting in FGR and other pregnancy complications [52]. Our findings indicate that ADF in the context of a high-fat diet may limit placental vascular connectivity, thus elevating the risk of FGR.

The nutrient transporters play a pivotal role within the placenta, primarily facilitating the translocation of essential nutrients to support fetal development and growth [53,54,55]. If the functionality of these transport proteins is compromised, it may result in restricted fetal growth [56]. Glucose is the primary energy substrate crucial for fetal growth and development [57], and its transfer from maternal to fetal circulation across the placenta primarily depends on the sole carrier family 2 of transporters [53]. Essential amino acids, which cannot be synthesized by either the placenta or the fetus, must be transported from maternal circulation through the placenta to ensure the normal growth and development of both the placenta and the fetus. In the later stages of pregnancy, the placenta predominantly expresses three subtypes of the amino acid transport system A: *Slc38a1*, *Slc38a2*, and *Slc38a4* [58]. Research by Farley et al. has identified that in placentas from obese groups, the expression of *Slc38a4* is significantly reduced, whereas no significant differences are observed in the expression of *Slc38a1* and *Slc38a2* [59]. In maternal circulation, lipids primarily exist as triglycerides, phospholipids, and cholesterol. Triglycerides must first be hydrolyzed into free fatty acids (FFAs) by placental triglyceride lipase [60]. Once hydrolyzed, these FFAs are transported across the placenta by fatty acid transporters, ensuring that the fetus receives adequate energy and lipid-derived nutrients essential for growth and development [55]. Fatty acid-binding protein (FABP) is the primary transporter for free fatty acids (FFAs). A study from Canada reported a downregulation in the protein expression levels of FABP3 in the placentas of obese pregnant women, indicating that maternal obesity negatively impacts placental fatty acid uptake [61]. In placentas of fetuses with growth restriction, the activity of several nutrient transport proteins is reduced, indicating that modifications in placental nutrient transport may directly contribute to fetal growth abnormalities [56]. Our research findings indicate that the ADF regimen in obese mice results in a diminished capacity of the placenta to transport amino acids, glucose, and fatty acids, which may lead to fetal growth restriction.

Type I collagen is crucial for providing structural support, ensuring both the integrity and functionality of the placenta. In conjunction with type I collagen, *Col3α1* contributes to the maintenance of tissue elasticity and strength. The α5 chain of type IV collagen, encoded by *Col4α5*, constitutes the baS.Eent membrane in the placenta, facilitating cell adhesion [62]. In our study, we find that TRF mitigates the negative impact of a high-fat diet on the expression levels of *Col3α1* and *Col4α5*, thereby preserving the structural integrity of the placenta.

Prior research has identified elevated levels of inflammatory markers in both the plasma and placenta of obese mothers, including macrophage-associated markers such as IL-6, IL-10, IL-1β, and MCP-1, among others [63,64]. Additionally, significant alterations have been observed in endoplasmic reticulum stress markers, including GRP78, XBP1s, and CHOP [65,66]. These biomarker changes may contribute to the pathogenesis of fetal growth restriction (FGR) by inducing apoptosis and inflammatory responses. Experimental studies in mice show that TRF can reduce placental inflammation, preventing adverse fetal metabolic outcomes linked to maternal obesity during pregnancy [67]. In the current study, further examination of stress markers within the high-fat diet cohort reveals that TRF notably alleviates placental endoplasmic reticulum stress. Furthermore, TRF effectively suppresses the placental inflammatory response triggered by a high-fat diet.

The results demonstrate that TRF alleviates endoplasmic reticulum stress in the placenta and upregulates the gene expressions of placental nutrient transporters and angiogenic factors, thereby counteracting the adverse effects of a high-fat diet on placental function and embryonic development. Conversely, ADF exacerbates the negative impacts of a high-fat diet. These findings suggest that TRF may serve as a more effective intervention strategy to enhance the metabolic health of obese mothers and improve placental function, thereby fostering healthy fetal development. However, this study has several limitations that should be acknowledged. First, the duration of the high-fat diet treatment in mice (30 days before mating) was not enough to induce obvious obesity. Furthermore, there are many different kinds of intermittent fasting patterns [68], such as the 5:2 diet, complete alternate day fasting, calorie restriction, and different kinds of time-restricted eating, but the study only compared two dietary patterns: alternate-day fasting and time-restricted eating. While this design provides insight into the direct effects of these two approaches, it excludes other potential fasting interventions, limiting a comprehensive understanding of how different dietary strategies impact obesity, placental function, and fetal development. Moreover, the obesity model used in the study was relatively singular, relying solely on a high-fat diet to induce obesity in mice. This may not adequately represent the diversity of human obesity, and the findings may only be applicable to obesity conditions induced by high-fat diets rather than other forms of obesity. Lastly, the study focused on fetal development and placental function without conducting long-term follow-up on postnatal health outcomes, such as the weight, metabolic health, or disease susceptibility of offspring. Research on the effects of intermittent fasting during pregnancy on offspring is limited [69]. As a result, the long-term effects of dietary interventions on fetal growth, particularly their impact on health in adulthood, remain unclear.

## 5. Conclusions

**Figure 5:**
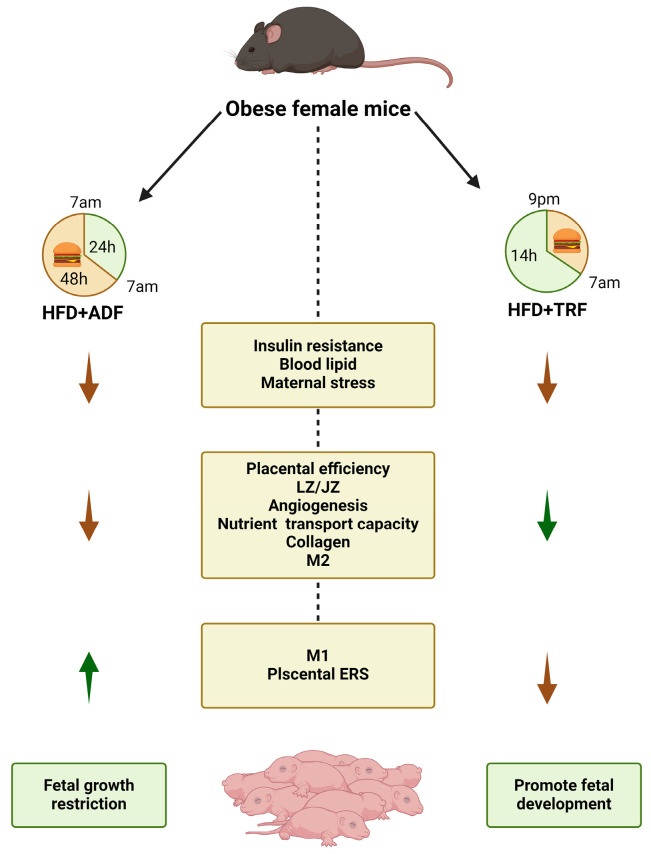
Effects of ADF and TRF on the fetus of obese mice during pregnancy. Both ADF and TRF have been shown to effectively reduce insulin resistance and lipid levels in obese pregnant female mice. Moreover, TRF ameliorated maternal obesity-related inflammatory responses and ER stress and improved placental function and fetal development. In contrast, ADF significantly decreases placental weight, leading to a diminished nutrient transport capacity and increased incidence of FGR.

In conclusion, our research demonstrates that TRF markedly enhances fetal weight in mice by mitigating maternal metabolic disorders caused by a high-fat diet, probably by optimizing placental function. Conversely, an ADF regimen leads to a reduction in the placental labyrinth zone area, decreased expression of placental nutrient transporters, heightened placental inflammation, and reduced expression of collagen proteins, culminating in fetal growth restriction (Figure 5). These findings highlight the critical role of dietary regimens during pregnancy, offering novel insights into how dietary interventions, particularly those associated with intermittent fasting, influence obesity and fetal development during gestation. This research not only deepens our understanding of the mechanistic role of dietary interventions during pregnancy but also suggests new strategies for addressing obesity and metabolic disorders during gestation. These findings could inform the development of targeted nutritional guidelines to improve pregnancy outcomes and maternal-fetal health.

## Data Availability

The data sets used and/or analyzed during this study are available on reasonable request from the corresponding author. Some of the data involve unpublished patent applications or proprietary information provided by commercial partners, and therefore cannot be made public.

## Supplementary Materials

The following supporting information can be downloaded at: https://www.mdpi.com/article/10.3390/nu17010025/s1. Table S1: Primers used in qPCR, related to VEGFα and Figure 4A. Table S2: Primers used in qPCR, related to Figure 4B. Figure S1: (**A**) The weight of the mice at 8 weeks of age at the start of the experiment. ND (*n* = 25), HD (*n* = 75). (**B**) Weight after one month of a normal diet (ND) and one month of a high-fat diet (HD). ND (*n* = 25), HD (*n* = 75). Data are expressed as the mean ± S.E. * *p* < 0.05, ** *p* < 0.01 and *** *p* < 0.001.

## Abbreviations

TRF	Time-restricted feeding
ADF	Alternate-day fasting
HFD	High-fat diet
NA	chow diet-ad libitum
HA	HFD-ad libitum
HI	HFD-ADF
HT	HFD-TRF
FGR	Fetal growth restriction
