# Supplementary material for: Comparative Impact of Alternate-Day Fasting and Time-Restricted Feeding on Placental Function and Fetal Development in Maternal Obesity
Source: Nutrients. 2024 Dec 25;17(1):25. doi: 10.3390/nu17010025 (PMC11723168; doi:10.3390/nu17010025)
Supplement: Supplementary file 1 [file nutrients-17-00025-s001.zip › nutrients-3333508-supplementary.pdf]

Comparitive impact of alternate-day fasting and time-restricted feeding on placental function and fetal development in maternal obesity mice anuscript
Siyuan Liu

Supplementary Materials

Supplementary Table S1

Gene	Forward Primer Sequence (5' -> 3')	Reverse Primer Sequence (5' -> 3')
β-Actin	GGCTGTGCTGTCCCTGTA	GGGCATAACCCTCGTAGAT
GAPDH	CTCAGTTGCTGAGGAGTCCC	ATTCGAGAGAAGGGAGGGCT
VEGFα	CTGTAACGATGAAGCCCTGGAG	TGGTGAGGTTGATCCGCAT
Slc2a1	CTCTGTCGGCCTCTTTGTTAAT	CCAGTTTGGAGAAGCCCATAAG
Slc2a3	TCATCAATGCACCTGAGACAATC	GTCCCTCACTTGGTAGGTCTT
Slc2a4	GTGACTGGAACACTGGTCCTA	CCAGCCACGTTGCATTGTAG
Slc2a5	CCAATATGGGTACAACGTAGCTG	GCGTCAAGGTGAAGGACTCAATA
Slc5a1	ATGCGGCTGACATCTCAGTC	ACCAAGGCGTTCCATTCAAAG
Slc1a1	CAGCAGTACAAAACCAAGCG	GACAAGTCCAAAGACGAGG
Slc7a1	CTCCTCGCTGGTTCTTCTGG	CCAAGGCTCGTGGTTTTTGG
Slc38a1	CGAACCAGGATGGAGACAAG	GCAAGCCCAGTCGTAGATG
Slc38a2	CCTTGGGCTTTCTTATGC	GAGGAGGTGAACGGAGTA
Slc38a4	GGTATCTTGGGCTTGTCC	CCTTCCTTGGCTGTCTTC
Slc27a1	TCTGTTCTGATTCGTGTTCGG	CAGCATATACCACTACTGGCG
Slc27α6	GTGGTTTTAGGGTTAGYGGGTTTTAG	AACCRAAAATAAACAAACAACACTCC
FABP3	AGTCACTGGTGACGCTGGACG	AGGCAGCATGGTGCTGAGCTG
FABP4	TTGGTCACCATCCGGTCAGA	CCTGTCGTCTGCGGTGATTT
Col1α1	GCTCCTCTTAGGGGCCACT	CCACGTCTCACCATTGGGG
Col1α2	GTAACTTCGTGCCTAGCAACA	CCTTTGTCAGAATACTGAGCAGC
Col3α1	CTGTAACATGGAAACTGGGGAAA	CCATAGCTGAACTGAAAACCACC
Col4α5	GTCCACCAGGTACAGAAGGTC	CTCCTTTCAAACCAGGTAAGCC

Supplementary Table S1: Primers used in qPCR, related to ***VEGFα*** **and** Fig 4A.

Supplementary Table S2

Gene	Forward Primer Sequence (5' -> 3')	Reverse Primer Sequence (5' -> 3')
F4/80	ATTCCACGCCAATTCATCGT	CAGGTCTCTTTGGAGTCAGC
Mcp1	ACCTGCTGCTACTATTCAC	CATTCAAGGTGCTGAAGAC
Tnf-α	CGCTGCTGTCTGCTTCA	CCTGGTCCTGGTTCACTC
Il-1β	AGGCTTCCTTGTGCAAGTGT	CATTGGAAGTTGGGGTAGGA
Il-6	GCCAGAGTCATTCAGAGCAA	CATTGGAAGTTGGGGTAGGA
Arg1	CATATCTGCCAAGGACATCG	ATTCCCAGCTTGTCCACTTC
Mgl1	CTGGATCCTGGTGTCTTGGT	AGGTGGGTCCAAGAGAGGAT
Il-10	AATCCCTTTGATTTTGCC	GTGCCTTATCCTACAGTATGTG
iNOS	ATCTTGGAGCGAGTTGTGGATTGTC	TAGGTGAGGGCTTGGCTGAGTG
Mrc2	ACCAACACTGGGACCTGTCAC	CGAAGAACACAGCCTTCTCCT
Chop	GAGGTGGAGACCACACGGCG	AAGCCGAGCCCTCTCCTGGT
XBP1s	GCTGAGTCCGCAGCAGGT	CTGGGTCCAAGTTGTCCAGAAT
GRP78	GCATCACGCCGTCGTATGT	ATTCCAAGTGCGTCCGATGAG
Perk	TTCCCAACTAACCCGAAGGC	ATTCGTCAGACCCCACCAAC
ATF6	AGCGCCCAAGACTCAAACC	CTGTATGCTGATAATCGACTGCT
IRE1	CTCCTCTGTCTGCATCCACCA	ACGGTGGTCGGTGTGTTGTC

Supplementary Table S2: Primers used in qPCR, related to Fig 4B.

Supplementary Figure S1

A **B**

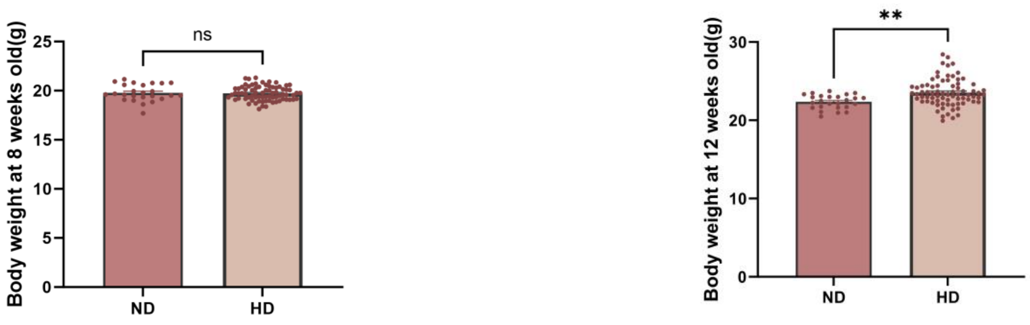

Supplementary Figure S1. A: The weight of the mice at 8 weeks of age at the start of the experiment. ND (N=25), HD(N=75). B: Weight after one month of a normal diet (ND) and one month of a high-fat diet (HD).ND (N=25), HD (N=75). Data are expressed as the mean ± S.E. *P < 0.05, **P < 0.01 and ***P < 0.001.
